# Hope mediates the association between anxiety and caregivers’ preparedness in schizophrenia: a cross-sectional study

**DOI:** 10.3389/fpubh.2025.1652448

**Published:** 2025-12-17

**Authors:** Yakun Liu, Weiyu Teng, Fei Teng, Guiyuan Zou

**Affiliations:** 1 Department of Healthcare Respiratory, Shandong Provincial Hospital Affiliated to Shandong First Medical University, Jinan, Shandong, China; 2 Department of Nursing, Shandong Mental Health Center, Shandong University, Jinan, Shandong, China; 3 Department of Psychiatric, Shandong Mental Health Center, Shandong University, Jinan, Shandong, China

**Keywords:** anxiety, hope, preparedness, caregivers, schizophrenia, mediation

## Abstract

**Background:**

This study investigated the level of preparedness of caregivers of patients with schizophrenia and explored hope as a mediator between anxiety and caregivers’ preparedness.

**Methods:**

This correlational cross-sectional study included 375 caregivers of patients with schizophrenia. Participants completed a self-administered questionnaire on preparedness, anxiety, and hope. Descriptive analysis, Pearson’s correlations, and hierarchical linear regression modeling were performed to examine the associations between anxiety, hope, and caregivers’ preparedness using SPSS 22.0. The PROCESS v3.3 macro was used to analyze the mediating effect of hope.

**Results:**

Caregivers’ preparedness, anxiety, and hope scores were (20.79 ± 5.25), (10.38 ± 4.56), and (36.01 ± 5.25), respectively. Anxiety was negatively correlated with hope and caregivers’ preparedness (*r* = −0.478, *p* < 0.001; *r* = −0.290, *p* < 0.001), and hope was positively correlated with caregivers’ preparedness (*r* = 0.513, *p* < 0.001). Hope partially mediated the relationship between anxiety and caregivers’ preparedness, accounting for 24.8% of the total effect.

**Conclusion:**

Anxiety among caregivers of patients with schizophrenia influences their preparedness and is mediated by hope. Enhancing hope among caregivers can reduce the impact of low preparedness caused by high anxiety.

## Introduction

Schizophrenia, one of the most prevalent functional psychotic disorders, is characterized by illusions, hallucinations, thought disturbances, and affective and volitional behavior disturbances. These symptoms affect both the patients and their caregivers, imposing substantial emotional strain and creating practical challenges for family members. Several studies have examined the caregiving role of family members in the lives of patients with schizophrenia and recognized that caring for these patients requires continuous vigor, relevant knowledge and skills, empathy, and financial capacity, as well as seriously affect their daily lives (1–3). Furthermore, early onset and chronic schizophrenia affects the lives of patients who could manifest social and relational disabilities and require years of caregiving. In China, most patients with schizophrenia choose to return to their families after receiving treatment during the acute phase, and their family members, as the main caregivers, provide continuous rehabilitation exercises and home care. Family caregivers play a vital role in meeting all aspects of patients’ care demands, including their physiological, cognitive, and emotional needs, and face challenges, such as care ability and psychology (4, 5). However, family caregivers, especially those assuming the caregiver role for the first time, are typically uncertain about the patient’s disease, have inadequate knowledge of psychotic disorders, and perceive an inability to discharge their caregiving function (6). Although caregivers feature in the course of treatment and recovery, those who do not receive appropriate support services and lack care knowledge and skills often feel unprepared to perform their role and experience a greater burden (7, 8). Prior studies on caregiver preparedness have focused on patients with cancer or dementia (9–11). This study focuses on extending this stream of research to the context of schizophrenia.

Caregivers’ preparedness is a multidimensional and dynamic construct, defined as “how ready caregivers believe they are for the tasks and stresses of caregiving” (12). Researchers have described the beneficial effects of caregivers’ preparedness (13–15). Greater preparation leads to better patient-reported outcomes and quality of life for caregivers. However, insufficient preparation has a negative impact on family dynamics and structure, thus affecting the capacity of family members to cope with caregiving-related demands effectively. A considerable body of research focuses on demographic comparisons of caregiver preparedness in the current healthcare context (13, 16, 17). For example, being less prepared is more common among men (18), the less educated (19), and those in inadequate financial situations (20). These findings underscore the need to develop a better understanding of the determinants of caregivers’ preparedness, thereby enabling targeted interventions in high-risk populations. Nevertheless, research specifically examining caregivers’ preparedness for patients with schizophrenia and the influencing factors has not yet been reported.

Caregiving is burdensome, with most caregivers experiencing sustained high levels of psychological distress (21). Furthermore, psychological factors are increasingly being recognized as critical determinants of caregiver preparedness. Among these factors, anxiety and hope are two contrasting yet interconnected emotional and cognitive constructs that may play pivotal roles in shaping caregivers’ preparedness.

Anxiety is a prominent negative emotional response to caregiving stressors and is highly prevalent among caregivers of patients with schizophrenia, with reported rates ranging from 29 to 41.4% (22–24). Studies have shown that anxiety not only negatively affects caregivers’ social function, economic status, physical health, and quality of life but also correlates with poorer patient-reported outcomes (21, 22, 25). Dionne-Odom et al. (26) found an association between low caregivers’ preparedness and greater anxiety among patients with cancer, suggesting that anxiety may erode caregivers’ sense of preparedness by consuming cognitive resources, increasing perceived burden, and impairing problem-solving abilities, all of which are critical for effective caregivers’ preparedness. However, research on the relationship between anxiety and caregivers’ preparedness specifically among patients with schizophrenia remains limited.

Hope, as a positive source of support for addressing caregiving stressors, has recently garnered increasing attention (27, 28). Hope is recognized as an intentional, highly personalized, and multidimensional dynamic process central to human life and oriented towards the future (29). Several studies have revealed relationships between hope and various factors, such as happiness, quality of life, problem-solving coping strategies, and management of loss, sadness, pain, and loneliness (30, 31). For caregivers, hope may enhance preparedness by fostering a sense of control, providing motivation to acquire caregiving skills, buffering the negative impact of stressors, and ultimately transforming how families navigate the challenges of caring for a loved one with a chronic illness (28, 32). Emerging research has highlighted the beneficial effects of hope in terms of disease management among primary caregivers of patients with schizophrenia (33, 34). However, its specific role in shaping caregivers’ preparedness remains understudied.

The rationale for examining anxiety and hope simultaneously lies in their conceptual and empirical interconnections, as well as their complementary roles in influencing caregiver outcomes. Previous studies have consistently demonstrated an inverse association between anxiety and hope. Higher anxiety corresponds to diminished hope, reflecting a potential dynamic in which negative emotional states may deplete positive cognitive resources, and conversely, positive cognitive orientations may mitigate psychological distress (18, 35). This interplay is particularly significant within the framework of caregivers’ preparedness. Anxiety may erode preparedness through its deleterious impact on cognitive functioning and emotional regulation, whereas hope can counteract such effects by fostering resilience and facilitating goal-directed behavior (36, 37). Furthermore, preliminary evidence has indicated that hope may mediate the relationship between stress-related factors and caregiver outcomes. For example, Liu et al. (38) demonstrated that hope can partially mediate the association between disease uncertainty and caregivers’ preparedness among family members of patients with heart failure, suggesting that positive cognitive resources, such as hope, may explain how negative experiences translate into variations in preparedness. Considering these research gaps and interconnections, we hypothesized that hope mediates the relationship between anxiety and caregivers’ preparedness of patients with schizophrenia. To date, no study has validated this association in this population.

Building upon the hypothesized mediating role of hope between anxiety and caregivers’ preparedness, this study aims to systematically investigate these dynamics in the context of schizophrenia caregiving. The primary objective was to investigate the level of caregivers’ preparedness. Subsequently, the study examines the interrelationships between anxiety, hope, and caregivers’ preparedness. Ultimately, it aims to test the specific mediating pathway of hope in the relationship between anxiety and caregivers’ preparedness.

## Materials and methods

### Ethical considerations

This descriptive, correlational, cross-sectional study was approved by the Ethics Committee of Shandong Mental Health Center (No. 2021R07). All participants were informed of the purpose of the study, assured that their data would be kept confidential, and provided signed informed consent before participating.

### Participants and procedure

Convenience sampling was used to recruit caregivers of patients with schizophrenia at a tertiary psychiatric hospital in Shandong Province, China, between October 2016 and December 2021. We selected caregivers in accordance with Martinez-Martin et al. (39) definition: “a person who is not a professional caregiver, who lives with or close to the assisted patient and is directly involved in the treatment and caring of the patient’s health problem.” The inclusion criteria were as follows: (1) being the primary family caregiver for a patient diagnosed with schizophrenia by a psychiatrist according to *International Classification of Diseases-10* (ICD-10) schizophrenia criteria, (2) being aged 18 years or older, (3) being involved in the caregiving experience for at least 1 year, (4) providing valid informed consent from themselves (caregivers) and their assisted patients, and (5) having no other severe physical diseases. Family caregivers were excluded if they: (1) experienced mental illness or (2) had language difficulties or could not cooperate with the survey.

### Data collection

We implemented a series of measures to ensure methodological consistency. All data were collected during face-to-face interviews conducted by uniformly trained investigators on the research team. The researcher introduced the purpose, significance, filling method, and time spent on the study and invited the participants to sign the informed consent form and complete the paper-based questionnaire. The questionnaires were primarily self-administered; however, for participants who experienced difficulties in independent completion (e.g., because of literacy barriers or physical limitations), investigators provided standardized assistance by reading questions aloud and recording responses as reported. Throughout the process, all participants were explicitly assured of the anonymity of their responses and the strict confidentiality of their personal information. After each participant completed the survey, the investigators immediately checked their responses on-site and asked them to complete the questionnaire if anything was omitted. The questionnaires were uniformly numbered and double-entered, excluding any with logical inconsistencies and evident irregularities. No deletion or imputation was performed because no data were missing. A total of 406 questionnaires were distributed, and 375 valid questionnaires were obtained, with an effective recovery rate of 92.4%. The most frequent reasons for incomplete surveys were unwillingness to participate in face-to-face interviews and being too busy.

### Sample size

We calculated the sample size using the following formula: *N* = (μα S/*δ*)2 = 221 (where μα = 1.96, and the S/δ value = 7.58 according to the pilot study). Considering a 20% nonresponse rate, we obtained a minimum sample size of 265. Our study employed sufficient participants to ensure robust statistical power, with a sample size of 375, meeting the recommendations.

### Participant characteristics

Data were collected on participants’ sociodemographic and clinical variables, including gender, age group, years of education, financial status (Considering your household’s current income, expenses, and savings, which of the following best describes your financial situation?), number of hospitalizations, perceived disease severity (Overall, to what extent do you think the disease interferes with his/her normal life?), mental health knowledge (How familiar are you with the topics related to mental health?), relationship with the patient, and caregiving experience (Before caring for patients with schizophrenia, have you had any experience caring for others?).

The Preparedness for Caregiving Scale, an eight-item scale included in the Family Care Inventory, was used to assess caregivers’ preparedness (40). Each item was rated on a 5-point scale ranging from 0 (not at all prepared) to 4 (very well prepared). The items were summed, with higher scores indicating higher levels of caregiver preparedness. A previous study using a sample of Chinese nurses reported satisfactory validity and reliability (41), and Cronbach’s *α* was 0.96 in the present study.

The Generalized Anxiety Disorder Scale (GAD-7) is a brief self-report scale used for generalized anxiety screening and symptom severity assessment (42). The items captured how often the participants were bothered by feelings associated with anxiety during the preceding two-week period. Seven items were scored from 0 (not at all) to 3 (almost every day), with total scores ranging from 0 to 21. Higher scores indicated more severe anxiety symptoms. The GAD-7 has shown good psychometric properties in previous studies on Chinese general and adolescent populations (43, 44), and Cronbach’s *α* was 0.82 in the present study.

The Herth Hope Index (HHI) comprises 12 items (developed by Herth) and is widely used to measure hopefulness at the individual level (45, 46). Each item was scored on a 4-point scale ranging from 1 (strongly disagree) to 4 (strongly agree). Responses were summed to obtain overall scores, which ranged from 12 to 48. Higher scores indicated greater hopefulness. The Chinese version of the HHI has demonstrated high reliability and validity (47), and Cronbach’s *α* was 0.87 in the present study.

### Statistical analysis

Statistical analyses were performed using SPSS 22.0. Descriptive statistics were used to describe participants **’** sociodemographic and clinical characteristics. Differences between participants’ preparedness based on their sociodemographic and clinical characteristics were assessed using a t-test or analysis of variance (ANOVA). Pearson’s *r* was used to examine the relationships between caregivers’ preparedness, anxiety, and hope. Hierarchical multiple regression analysis was performed with caregivers’ preparedness as the dependent variable to address potential confounders. Years of education, financial status, number of hospitalizations, mental health knowledge, perceived disease severity, and caregiving experience were entered as control variables in Block 1, anxiety in Block 2, and hope in Block 3. Finally, the mediating role of hope between anxiety and caregivers’ preparedness was explored using the PROCESS macro (Model 4) of SPSS 22.0, following Hayes (48, 49). The analysis used 5,000 bootstrapped samples and 95% bias-corrected confidence intervals to assess the statistical significance of the mediated models and the magnitude of the mediated effect. For all analyses, *p* < 0.05 was considered statistically significant.

## Results

### Diagnostic tests for model assumptions

Before conducting the empirical analysis, we tested the normality assumption and conducted multicollinearity diagnostics. Normality was assessed using the Kolmogorov–Smirnov test (for sample sizes >50). The results indicated that all continuous variables met the assumption of normality (*p* > 0.05). Multicollinearity was evaluated using variance inflation factors, for which all values were far below the critical value of 5, with a mean of 1.20 and a maximum of 1.47, indicating no significant multicollinearity in our data.

### Description of sample characteristics

As Table 1 shows, the sample comprised 201 (53.6%) men and 174 (46.4%) women. Almost half of the caregivers were between 36 and 50 years of age (48.0%). Among the participants, 29.9% had less than 9 years of education, 39.5% had 9–12 years, and 30.7% had more than 12 years. Regarding family financial status, 45.9% of the participants reported their status as insufficient. In terms of their relationship with the patients, 47.5% were parents, 23.2% were children, and 26.9% were spouses. Perceived disease severity ranged from mild to severe at 31.5%, 41.0%, and 27.5%, respectively. Regarding mental health knowledge, 38.9% of participants were unfamiliar, 44.0% had a basic level of familiarity, and 17.1% were very familiar. Among the caregivers, 69.6% had no prior caregiving experience. Only 28.0% of the sample had been hospitalized once.

**Table 1:** Description of socio-demographic and clinical characteristics and univariate analysis of caregivers’ preparedness.

Variable	*n* (%)	x̄ ± s	t/F	*p*
Sex			0.001	0.980
Male	201 (53.6)	20.80 ± 5.04		
Female	174 (46.4)	20.79 ± 5.49		
Age group			0.360	0.698
≤35 years old	123 (32.8)	20.47 ± 5.22		
36 ~ 50 years old	180 (48.0)	20.92 ± 5.31		
≥51 years old	72 (19.2)	21.04 ± 5.21		
Years of education			4.391	0.013
≤9 years	113 (30.1)	19.71 ± 5.17		
9 ~ 12 years	147 (39.2)	20.86 ± 5.57		
≥12 years	115 (30.7)	21.76 ± 4.73		
Financial status			4.132	0.017
Insufficiency	172 (45.9)	20.11 ± 5.11		
Balance	123 (32.8)	20.87 ± 5.07		
Rich	75 (21.3)	22.14 ± 5.61		
Hospitalization times			20.499	0.000
First-episode psychosis	105 (28.0)	19.04 ± 4.21		
Recurrence	270 (72.0)	21.47 ± 5.46		
Perception of disease severity			5.708	0.004
Mild	118 (31.5)	21.60 ± 4.46		
Moderate	154 (41.1)	21.14 ± 5.60		
Severe	103 (27.5)	19.36 ± 5.31		
Knowledge of mental health			11.554	0.000
Unfamiliar	146 (38.9)	19.44 ± 5.36		
Basic familiar	165 (44.0)	21.13 ± 4.94		
Very familiar	64 (27.5)	23.02 ± 4.95		
Relations with patients			1.157	0.326
Parents	178 (47.5)	21.14 ± 5.17		
Children	87 (23.2)	20.48 ± 4.70		
Spouses	101 (26.9)	20.69 ± 5.84		
Others	9 (2.4)	18.11 ± 4.51		
Caring experience			4.065	0.045
No	261 (69.6)	20.39 ± 4.97		
Yes	114 (30.4)	21.71 ± 5.77		

### Variable scores and correlations

As Table 2 shows, participants’ mean scores for preparedness, anxiety, and hope were (20.79 ± 5.25), (10.38 ± 4.56), and (36.01 ± 5.25), respectively. Caregivers’ preparedness scores differed significantly depending on years of education, financial status, number of hospitalizations, mental health knowledge, perceived disease severity, and caregiving experience. No significant differences were found between caregivers’ preparedness scores and other sociodemographic characteristics. The correlation analysis showed that caregivers who reported higher levels of anxiety displayed less hope and preparedness (*r* = −0.478, *p* < 0.001; *r* = −0.290, *p* < 0.001). The relationship between hope and caregivers’ preparedness was significant and positive (*r* = 0.513, *p* < 0.001).

**Table 2:** Mean, standard deviations (SD) and correlation of variables (r).

Variables	x̄ ± s	Caregivers’ preparedness	Hope	Anxiety
Caregivers’ preparedness	20.79 ± 5.25	1		
Hope	36.01 ± 6.00	0.513*	1	
Anxiety	10.38 ± 4.56	−0.478*	−0.290^*^	1

* *p* < 0.001.

### Regression model

We performed a regression-based mediation analysis using SPSS PROCESS macro Model 4 to test the hypothesis that hope mediates the relationship between anxiety and caregivers’ preparedness (48). This analysis for simple and parallel mediation utilized the bootstrapping method to estimate the direct and indirect effects. Indirect effects were subjected to follow-up bootstrap analyses using 5,000 bootstrap samples and 95% bias-corrected confidence intervals. Following Aiken et al. (49) recommendations, the predictor variables were centered. Years of education, financial status, number of hospitalizations, mental health knowledge, perceived disease severity, and caregiving experience were included as control variables. Table 3 summarizes the analysis results.

**Table 3:** Hierarchical linear regression analysis results.

Variables	Caregivers’ preparedness		
	Step 1 (*β*)	Step 2 (*β*)	Step 3 (*β*)
Block 1			
Years of education	0.118*	0.121**	0.080*
Financial status	0.081	0.062	0.027
Hospitalization times	0.415***	0.350***	0.313***
Knowledge of mental health	0.237***	0.182***	0.135***
Perception of disease severity	−0.328***	−0.189***	−0.169***
Caring experience	0.223***	0.177***	0.313***
Block 2			
Anxiety		−0.387***	−0.298***
Block 3			0.356***
Hope			
F	20.001***	31.462***	42.966***
*R*^2^	0.246	0.375	0.484
△*R*^2^	0.246	0.129	0.109

*p < 0.05; **p < 0.01; ***p < 0.001.

The hierarchical linear regression analysis (Table 3) showed that all control variables, except family financial status, were significantly associated with caregivers’ preparedness in step 1 (*R*^2^ = 0.246). Anxiety was a significant negative predictor of caregivers’ preparedness in step 2, explaining 12.9% of the variance. In step 3, hope positively predicted caregivers’ preparedness, accounting for 10.9% of the variance. When hope was added the regression model, the relationship between anxiety and caregivers’ preparedness was significantly reduced. Finally, education, number of hospitalizations, disease-related knowledge, perceived disease severity, caregiving experience, anxiety, and hope were associated with caregivers’ preparedness, explaining 48.4% of the variance.

When hope was added to in step 3, the regression coefficients for anxiety (from *β* = − 0.387 to *β* = − 0.298, *p* < 0.001) decreased. Following Baron and Kenny’s mediation test, we inferred that hope partially mediated the relationship between anxiety and caregivers’ preparedness. The bootstrapping results (Table 4) showed that the path coefficient of the indirect effect of anxiety on caregivers’ preparedness was −0.1364 (95% CI: −0.2014, −0.0860), supporting the assertion that hope is a partial mediator. The indirect effect of anxiety on caregivers’ preparedness through hope accounted for 24.8% of the total effect.

**Table 4:** Hope’s mediation between anxiety and caregivers’ preparedness.

Mediation model hypothesis	Effect	Point estimate	SE	95% CI	
				LL	UL
Anxiety	Total effect	−0.5496	0.0523	−0.6526	−0.4467
Hope	Direct effect	−0.4132	0.0490	−0.5097	−0.3168
Caregivers’ preparedness	Indirect effect	−0.1364	0.0293	−0.2014	−0.0860

## Discussion

This study addresses two critical gaps in schizophrenia caregiving research: the under-researched area of caregivers’ preparedness and the unexamined mediating role of hope between anxiety and caregivers’ preparedness. To the best of our knowledge, it is the first study to investigate these relationships. The results showed that the mean caregivers’ preparedness score was below the medium level. Furthermore, anxiety significantly and negatively predicted both hope and caregivers’ preparedness, while hope itself was positively correlated with caregivers’ preparedness. Crucially, mediation analysis confirmed that hope partially mediates the relationship between anxiety and caregivers’ preparedness.

Caring for a family member is a highly stressful and demanding role. Caregivers’ preparedness, benefit-finding, and caregiving ability are key outcome indicators that have long been the focus of research in the caregiving field (50–52). In this study, the mean caregivers’ preparedness score was (20.79 ± 5.25), which is significantly lower than that reported for caregivers of patients without chronic psychiatric illnesses, indicating substantial room for improvement among caregivers of patients with schizophrenia (51–55). However, this score was higher than that reported for caregivers of patients with eating disorders (56). One possible explanation is that national attention to mental health, especially common mental illnesses, is increasing, thereby gradually enhancing mental health literacy among the general public (57, 58). Our study found significant associations between caregiver preparedness and years of education, financial status, caregiving experience, mental health knowledge, and number of hospitalizations, which is consistent with prior research (9–11, 51–55). Research shows that caregivers with higher education, better financial status, more mental health knowledge, more hospitalizations, and richer caregiving experience tend to show greater ability to actively seek medical information, handle crises, and adapt to their role, all of which are factors that may enhance caregivers’ preparedness. Furthermore, this study identified a negative correlation between perceived disease severity and caregivers’ preparedness. A plausible explanation is that the diagnosis and treatment of schizophrenia, as a major stressor, imposes a substantial psychosomatic burden on caregivers. Higher perceived disease severity correlates with heightened stress, necessitating more time and effort for coping, thereby increasing caregiving burden and impairing preparedness (59). However, this study had several limitations. First, key variables-including financial status, knowledge of mental health, and disease severity, were measured based on caregivers’ subjective reports. Although such measures capture meaningful lived experiences, they are susceptible to bias from factors such as mood or caregiving burden. For instance, financial status was categorized broadly, which lacks the precision of objective indicators like household income or socioeconomic indices. Similarly, knowledge of mental health was based on self-assessment rather than objective mental health literacy tests. Theoretical frameworks posit that insufficient knowledge may contribute to anxiety, the inverse relationship, whereby anxiety symptoms bias self-perceptions of knowledge, warrants equal consideration (60). Therefore, relying solely on self-reported measures may confound these constructs. The severity of disease reflects the extent to which caregivers believe the disease has affected the patient’s life. However, reliance on caregiver-reported severity may introduce bias owing to factors such as anxiety or unmeasured confounders. Future studies should incorporate objective measures, such as income brackets, standardized mental health literacy tests, and clinician-rated symptom scales (e.g., PANSS), to enhance validity and comparability. Second, the number of hospitalizations was used as a proxy for illness progression. While this metric reflects significant care-related stressors, it does not distinguish between clinical staging (e.g., prodromal vs. acute phases) or symptom severity at admission. While frequent hospitalizations may suggest disease recurrence or worsening, they do not fully capture individual symptom profiles or precise illness progression. Future studies should incorporate clinician-rated symptom scales (e.g., PANSS) to better delineate disease course and improve classification accuracy. Third, our measurement of caregiving experience was a dichotomized (yes/no) classification. While this may not capture variations in caregiving duration intensity, or daily caregiving hours, it provides a preliminary assessment of caregiver status. Future studies should incorporate detailed temporal and behavioral metrics (e.g., daily hours, total months) to better quantify caregiving burden.

Consistent with prior studies (10, 19, 61), we confirmed a significant negative association between anxiety and caregivers’ preparedness. Specifically, anxiety, which independently explained 12.9% of the variance in caregivers’ preparedness, can manifest as debilitating symptoms (e.g., diminished interest, impaired concentration, appetite loss, restlessness, despair, and suicidal ideation) (21–24, 62, 63) that directly erode functional capacities. This symptom burden compromises communication with healthcare providers and impedes the acquisition of disease knowledge (61), leading to a synergistic decline in caregiving ability, confidence, and engagement, ultimately resulting in markedly low preparedness. These findings suggest practical methods for improving caregivers’ preparedness. First, early screening for anxiety (e.g., using GAD-7) can facilitate timely mental health support. Second, psychoeducational programs incorporating coping strategies (e.g., mindfulness) and peer support groups may help alleviate anxiety symptoms and foster shared problem-solving. Integrating these strategies into tailored interventions could effectively reduce anxiety and thereby improve preparedness, ultimately benefiting both caregivers and patients.

Aligned with international caregiver studies (56, 64), we found a strong positive correlation between hope and caregivers’ preparedness. Hope serves as a pivotal psychological resource for caregivers facing chronic conditions like schizophrenia (34). The caregiving context, characterized by unpredictable challenges, relational losses, and life disruptions, often erodes hope (28). Conversely, hope helps caregivers construct and sustain meaning amid adversity. Empirically, caregivers with higher hope report lower psychosomatic stress, greater well-being, and a stronger sense of competence (65). They also exhibit more enthusiasm, motivation, and positive self-identity when confronting caregiving demands, which directly enhances caregivers’ preparedness (18, 65). Based on our findings, intervention designs should strategically integrate hope-enhancing components to target both the direct and indirect pathways identified in this study. Specifically, structured hope-focused programs could employ a multimodal approach. Cognitive-behavioral techniques could support caregivers in reframing negative appraisals of caregiving challenges (66, 67), while narrative therapy exercises could facilitate the reconstruction of personal resilience (68). These exercises emphasize past successes in overcoming caregiving obstacles to reinforce goal-directed thinking. Furthermore, psychoeducational modules focused on schizophrenia prognosis and caregiving skills can alleviate the critical hope-depleting factor of uncertainty by enhancing caregivers’ understanding of disease trajectories and equipping them with practical problem-solving tools (69, 70).

The most notable finding of this study was the mediating effect of hope on the relationship between anxiety and caregivers’ preparedness of patients with schizophrenia. This finding suggests that anxiety not only directly influenced caregiver preparedness but also indirectly influenced it via hope. Life-threatening situations, losses, and changes throughout life can affect a person’s level of hope. Motaghi et al. (27) found that one member’s disease affects the lives of everyone in the family, especially primary caregivers, thereby increasing anxiety and decreasing hope. Simultaneously, anxiety can reduce caregivers’ expectations of disease prognosis and medical decision-making, leading to lower levels of hope (71). Hope is related to factors such as well-being, quality of life, survival, problem-solving ability, and dealing with loss, tragedies, suffering, and loneliness (72). Individuals with more hope are inclined to adopt positive attitudes and coping strategies when faced with practical care problems. This mediating effect (24.8% of the total effect) highlights a critical intervention target: mitigating anxiety through hope-focused strategies rather than solely relying on anxiety-reducing techniques. For example, mindfulness programs could incorporate hope-building elements, such as reflecting on “small wins” in caregiving to counter anxiety-induced hopelessness (73, 74). Peer support interventions in which experienced caregivers share their journeys of maintaining hope amid anxiety may also foster vicarious learning and reduce feelings of isolation, both of which strengthen hope (75).

This study utilized a convenience sample from a single psychiatric hospital, which limits sample representativeness and potentially restricts generalizability to broader populations. Future research should use multicenter sampling across diverse regions to enhance population diversity and improve the representativeness of the findings. Second, our conclusions were based on cross-sectional data, which resulted in a one-time measurement of the variables. Third, social support and depression have been shown to significantly influence caregivers’ preparedness; however, we did not address this issue in our study. Therefore, future research should examine the associations between social support, depression, and caregiver preparedness. Finally, although this study clarified the importance of hope for caregiver preparedness, follow-up studies are needed to determine the effects of hope-based educational interventions on caregivers’ preparedness.

## Conclusion

The preparedness of caregivers of patients with schizophrenia shows room for improvement. This study found that caregivers’ preparedness was negatively related to anxiety but positively related to hope. This study also showed that hope could mediate the relationship between anxiety and caregivers’ preparedness. These findings call for healthcare professionals to take various measures to weaken anxiety and promote hope, both of which are beneficial for caregivers’ preparedness.

From a policy and practice perspective, these findings highlight the need to systematically integrate hope-focused support into caregiving services while offering practical guidance for clinical and community interventions. Healthcare systems and mental health organizations should train providers (e.g., psychiatrists, social workers, and nurses) to assess hope and anxiety among caregivers using validated tools to enable personalized interventions for high-risk groups (e.g., those with high anxiety or new caregivers) and implement hope-building programs tailored to caregivers of patients with schizophrenia, including psychoeducation on realistic care goals, peer success stories, and skill development, alongside anxiety-reducing strategies such as mindfulness, counseling, and support groups. Policymakers could fund community-based programs offering psychoeducation, peer mentorship, and counseling to sustain hope throughout caregiving, as hope’s far-reaching benefits-enhancing well-being, problem solving, and coping–may reduce burnout, improve care quality, and lessen healthcare burdens. Future research could strengthen the current evidence by using longitudinal designs to examine how anxiety, hope, and caregiver preparedness interact over time and whether sustained hope fosters long-term resilience, supporting the scalability of such programs. Finally, randomized controlled trials comparing hope-focused interventions (e.g., cognitive-behavioral versus peer support) are needed to refine evidence-based strategies to improve outcomes for caregivers and patients.

## Data Availability

The raw data supporting the conclusions of this article will be made available by the authors, without undue reservation.

## References

[1] Kamil SH Velligan DI. Caregivers of individuals with schizophrenia: who are they and what are their challenges? Curr Opin Psychiatry. (2019) 32:157–63. doi: 10.1097/YCO.0000000000000492, 30720485

[2] Schuster F Holzhüter F Heres S Hamann J. Caregiver involvement in psychiatric inpatient treatment - a representative survey among triads of patients, caregivers and hospital psychiatrists. Epidemiol Psychiatr Sci. (2020) 29:e129. doi: 10.1017/S2045796020000426, 32438939 PMC7264704

[3] Bademli K Lok N. Feelings, thoughts and experiences of caregivers of patients with schizophrenia. Int J Soc Psychiatry. (2020) 66:452–9. doi: 10.1177/0020764020916211, 32323603

[4] Huang C Lam L Plummer V Cross WM. Feeling responsible: family caregivers’ attitudes and experiences of shared decision-making regarding people diagnosed with schizophrenia: a qualitative study. Patient Educ Couns. (2021) 104:1553–9. doi: 10.1016/j.pec.2020.10.032, 33218782

[5] Chen L Zhao Y Tang J Jin G Liu Y Zhao X . The burden, support and needs of primary family caregivers of people experiencing schizophrenia in Beijing communities: a qualitative study. BMC Psychiatry. (2019) 19:75. doi: 10.1186/s12888-019-2052-4, 30786852 PMC6381607

[6] Shiraishi N Reilly J. Positive and negative impacts of schizophrenia on family caregivers: a systematic review and qualitative meta-summary. Soc Psychiatry Psychiatr Epidemiol. (2019) 54:277–90. doi: 10.1007/s00127-018-1617-8, 30349961

[7] Ducharme FC Lévesque LL Lachance LM Kergoat MJ Legault AJ Beaudet LM . “Learning to become a family caregiver” efficacy of an intervention program for caregivers following diagnosis of dementia in a relative. Gerontologist. (2011) 51:484–94. doi: 10.1093/geront/gnr014, 21383112

[8] Schumacher K Beck CA Marren JM. Family caregivers: caring for older adults, working with their families. Am J Nurs. (2006) 106:40–9. doi: 10.1097/00000446-200608000-00020 16905931

[9] Otto AK Ketcher D Heyman RE Vadaparampil ST Ellington L Reblin M. Communication between advanced Cancer patients and their family caregivers: relationship with caregiver burden and preparedness for caregiving. Health Commun. (2021) 36:714–21. doi: 10.1080/10410236.2020.1712039, 31910681 PMC9118123

[10] Dionne-Odom JN Azuero A Taylor RA Wells RD Hendricks BA Bechthold AC . Resilience, preparedness, and distress among family caregivers of patients with advanced cancer. Support Care Cancer. (2021) 29:6913–20. doi: 10.1007/s00520-021-06265-y, 34031751 PMC9733586

[11] Durepos P Ploeg J Akhtar-Danesh N Sussman T Orr E Kaasalainen S. Caregiver preparedness for death in dementia: an evaluation of existing tools. Aging Ment Health. (2020) 24:1671–80. doi: 10.1080/13607863.2019.1622074, 31144986

[12] Schumacher KL Stewart BJ Archbold PG. Conceptualization and measurement of doing family caregiving well. Image J Nurs Sch. (1998) 30:63–70. doi: 10.1111/j.1547-5069.1998.tb01238.x, 9549944

[13] Pucciarelli G Lyons KS Petrizzo A Ambrosca R Simeone S Alvaro R . Protective role of caregiver preparedness on the relationship between depression and quality of life in stroke dyads. Stroke. (2022) 53:145–53. doi: 10.1161/STROKEAHA.120.034029, 34496626

[14] Rha SY Park Y Song SK Lee CE Lee J. Caregiving burden and the quality of life of family caregivers of cancer patients: the relationship and correlates. Eur J Oncol Nurs. (2015) 19:376–82. doi: 10.1016/j.ejon.2015.01.004, 25795160

[15] Schumacher KL Stewart BJ Archbold PG. Mutuality and preparedness moderate the effects of caregiving demand on cancer family caregiver outcomes. Nurs Res. (2007) 56:425–33. doi: 10.1097/01.NNR.0000299852.75300.03, 18004189

[16] Nguyen HQ Haupt EC Duan L Hou AC Wang SE Mariano JD . Hospital utilisation in home palliative care: caregiver health, preparedness and burden associations. BMJ Support Palliat Care. (2022) doi: 10.1136/bmjspcare-2021-003455 PMC13724112 35078873

[17] Magasi S Buono S Yancy CW Ramirez RD Grady KL. Preparedness and mutuality affect quality of life for patients with mechanical circulatory support and their caregivers. Circ Cardiovasc Qual Outcomes. (2019) 12:e004414. doi: 10.1161/CIRCOUTCOMES.117.004414, 30636480

[18] Gutierrez-Baena B Romero-Grimaldi C. Predictive model for the preparedness level of the family caregiver. Int J Nurs Pract. (2022) 28:e13057. doi: 10.1111/ijn.13057, 35388583 PMC9285821

[19] Henriksson A Arestedt K. Exploring factors and caregiver outcomes associated with feelings of preparedness for caregiving in family caregivers in palliative care: a correlational, cross-sectional study. Palliat Med. (2013) 27:639–46. doi: 10.1177/0269216313486954, 23670720

[20] Hebert RS Dang Q Schulz R. Preparedness for the death of a loved one and mental health in bereaved caregivers of patients with dementia: findings from the REACH study. J Palliat Med. (2006) 9:683–93. doi: 10.1089/jpm.2006.9.683, 16752974

[21] Qiu D Li Y Wu Q An Y Tang Z Xiao S. Patient’s disability and caregiver burden among Chinese family caregivers of individual living with schizophrenia: mediation effects of potentially harmful behavior, affiliate stigma, and social support. Schizophrenia. (2023) 9:83. doi: 10.1038/s41537-023-00418-0, 38040711 PMC10692118

[22] Vadher S Desai R Panchal B Vala A Ratnani IJ Rupani MP . Burden of care in caregivers of patients with alcohol use disorder and schizophrenia and its association with anxiety, depression and quality of life. Gen Psychiatr. (2020) 33:e100215. doi: 10.1136/gpsych-2020-100215, 32656497 PMC7319701

[23] Yu Z Hu Y Tian W Zhu S Tang Y Xu D . Prevalence of depression, anxiety symptoms and related factors in family members of schizophrenic patients in a Shanghai community. Chin J Gen Pract. (2018) 17:587–90. doi: 10.3760/cma.j.issn.1671-7368.2018.08.003

[24] Zhou W Ma SC Cheng M Zhang XL Jing GJ Qu L . Relationship between perceived social support and anxiety among family caregivers of patients with schizophrenia: the mediating role of coping styles. Chin Nurs Res. (2021) 35:2090–5. doi: 10.12102/j.issn.1009-6493.2021.12.004

[25] Sharma R Sharma S Pradan S. Assessing caregiver burden in caregivers of patients with schizophrenia and bipolar affective disorder in Kathmandu medical college. J Nepal Health Res Counc. (2017) 15:258–63. doi: 10.3126/jnhrc.v15i3.18851, 29353899

[26] Dionne-Odom JN Demark-Wahnefried W Taylor RA Rocque GB Azuero A Acemgil A . The self-care practices of family caregivers of persons with poor prognosis cancer: differences by varying levels of caregiver well-being and preparedness. Support Care Cancer. (2017) 25:2437–44. doi: 10.1007/s00520-017-3650-7, 28247128 PMC5481472

[27] Sandström L Engström Å Nilsson C Juuso P. Experiences of suffering multiple trauma: A qualitative study. Intensive Crit Care Nurs. (2019) 54:1–6. doi: 10.1016/j.iccn.2019.07.006 31351691

[28] Hernandez M Barrio C Gaona L Helu-Brown P Hai A Lim C. Hope and schizophrenia in the Latino family context. Community Ment Health J. (2019) 55:42–50. doi: 10.1007/s10597-018-0354-5, 30506465 PMC6629030

[29] Snyder CR. Hope theory: rainbows in the mind. Psychol Inq. (2002) 13:249–75. doi: 10.1207/S15327965PLI1304_01

[30] Sari SP Agustin M Wijayanti DY Sarjana W Afrikhah U Choe K. Mediating effect of Hope on the relationship between depression and recovery in persons with schizophrenia. Front Psych. (2021) 12:627588. doi: 10.3389/fpsyt.2021.627588, 33633611 PMC7899985

[31] Garcia-Castro FJ Alba A Blanca MJ. Association between character strengths and caregiver burden: hope as a mediator. J Happiness Stud. (2020) 21:1445–62. doi: 10.1007/s10902-019-00138-2

[32] Adams K. Specific effects of caring for a spouse with dementia: differences in depressive symptoms between caregiver and non-caregiver spouses. Int Psychogeriatr. (2008) 20:508–20. doi: 10.1017/S1041610207006278 17937825

[33] Al-Rawashdeh S Alshraifeen A Rababa M Ashour A. Hope predicted quality of life in dyads of community-dwelling patients receiving hemodialysis and their family caregivers. Qual Life Res. (2020) 29:81–9. doi: 10.1007/s11136-019-02378-4, 31792798

[34] Duggleby W Lee H Nekolaichuk C Fitzpatrick-Lewis D. Systematic review of factors associated with hope in family carers of persons living with chronic illness. J Adv Nurs. (2021) 77:3343–60. doi: 10.1111/jan.14858, 33876845

[35] Naji GMA Isha ASN Alazzani A Saleem MS Alzoraiki M. Assessing the mediating role of safety communication between safety culture and employees safety performance. Front Public Health. (2022) 10:840281. doi: 10.3389/fpubh.2022.840281, 35359765 PMC8960200

[36] Saleem MS Isha ASNB Benson C Awan MI Naji GMA Yusop YB. Analyzing the impact of psychological capital and work pressure on employee job engagement and safety behavior. Front Public Health. (2022) 10:1086843. doi: 10.3389/fpubh.2022.1086843, 36620270 PMC9815146

[37] Naji GMA Isha ASN Mohyaldinn ME Leka S Saleem MS Rahman SMNBSA . Impact of safety culture on safety performance; mediating role of psychosocial Hazard: An integrated modelling approach. Int J Environ Res Public Health. (2021) 18:8568. doi: 10.3390/ijerph18168568, 34444314 PMC8394037

[38] Liu YM Sun DD Wang YF Gaofeng Z Qing Z . Mediating effect of caregiver hope level of famny members of patients with heart failure on disease uncertainty and caregiver readiness. Chin Med Herald. (2022) 19:164–8. doi: 10.20047/j.issn1673-7210.2022.07.037

[39] Martínez-Martín P Forjaz MJ Frades-Payo B Rusiñol AB Fernández-García JM Benito-León J . Caregiver burden in Parkinson’s disease. Mov Disord. (2007) 22:924–1060. doi: 10.1002/mds.21355, 17238193

[40] Archbold PG Stewart BJ Greenlick MR Harvath T. Mutuality and preparedness as predictors of caregiver role strain. Res Nurs Health. (1990) 13:375–84. doi: 10.1002/nur.4770130605, 2270302

[41] Liu YJ Wang M Dong XF. Reliability and validity of Chinese version of the caregiver preparedness scale in caregivers of stroke survivors. Chin J Pract Nurs. (2016) 32:1045–8. doi: 10.3760/cma.j.issn.1672-7088.2016.14.002

[42] Spitzer RL Kroenke K Williams JB Löwe B. A brief measure for assessing generalized anxiety disorder: the GAD-7. Arch Intern Med. (2006) 166:1092–7. doi: 10.1001/archinte.166.10.1092, 16717171

[43] Sun J Liang K Chi X Chen S. Psychometric properties of the generalized anxiety disorder Scale-7 item (GAD-7) in a large sample of Chinese adolescents. Healthcare. (2021) 9:1709. doi: 10.3390/healthcare9121709, 34946435 PMC8701121

[44] Ip H Suen YN Hui CLM Wong SMY Chan SKW Lee EHM . Assessing anxiety among adolescents in Hong Kong: psychometric properties and validity of the generalised anxiety Disorder-7 (GAD-7) in an epidemiological community sample. BMC Psychiatry. (2022) 22:703. doi: 10.1186/s12888-022-04329-9, 36376799 PMC9664827

[45] Herth K. Development and refinement of an instrument to measure hope. Sch Inq Nurs Pract. (1991) 5:39–51. 2063043

[46] Herth K. Abbreviated instrument to measure hope: development and psychometric evaluation. J Adv Nurs. (1992) 17:1251–9. doi: 10.1111/j.1365-2648.1992.tb01843.x, 1430629

[47] Chan KS Li HC Chan SW Lopez V. Herth hope index: psychometric testing of the Chinese version. J Adv Nurs. (2012) 68:2079–85. doi: 10.1111/j.1365-2648.2011.05887.x, 22111952

[48] Aiken LS West SG Reno RR. Multiple regression: Testing and interpreting interactions. Newbury Park, CA, USA: Sage (1991).

[49] Hayes AF. Introduction to mediation, moderation, and conditional process analysis: Methodology in the social sciences. New York, NY: The Guilford Press (2013).

[50] Geng XX Yang H Cao HL Cheng H Chen YJ. Research progress on preparedness for caregiving of patients with chronic diseases. Chin Nurs Res. (2020) 34:1954–9. doi: 10.12102/j.issn.1009-6493.2020.11.017

[51] Alam S Hannon B Zimmermann C. Palliative Care for Family Caregivers. J Clin Oncol. (2020) 38:926–36. doi: 10.1200/JCO.19.00018, 32023152

[52] Cheng ST Au A Losada A Thompson LW Gallagher-Thompson D. Psychological interventions for dementia caregivers: what we have achieved, what we have learned. Curr Psychiatry Rep. (2019) 21:59. doi: 10.1007/s11920-019-1045-9, 31172302 PMC6554248

[53] Lin H Lin R Yan M Lin L Sun X Wu M . Associations between preparedness, perceived stress, depression, and quality of life in family caregivers of patients with a temporary enterostomy. Eur J Oncol Nurs. (2024) 70:102557. doi: 10.1016/j.ejon.2024.102557, 38581900

[54] Uhm KE Jung H Woo MW Kwon HE Oh-Park M Lee BR . Influence of preparedness on caregiver burden, depression, and quality of life in caregivers of people with disabilities. Front Public Health. (2023) 11:1153588. doi: 10.3389/fpubh.2023.1153588, 37564425 PMC10409988

[55] Sun X Xu L Sheng L. Family hardiness among primary caregivers of breast cancer patients in Hunan Province: a cross-sectional study exploring the relationship with attachment and caregiver preparedness. Front Public Health. (2024) 12:1367029. doi: 10.3389/fpubh.2024.1367029, 39555037 PMC11563949

[56] Zhao YS Zheng MH Guo WD Gao SK Zhou S. Analysis of the current status and influencing factors of primary caregivers’ readiness for patients with eating disorders. Nurs Pract Res. (2024) 21:1517–22. doi: 10.3969/j.issn.1672-9676.2024.10.015

[57] Jiang GR Li DY Ren ZH Yan Y Wu X Zhu X . The status quo and characteristics of Chinese mental health literacy. Acta Psychol Sin. (2021) 53:182–98. doi: 10.3724/SP.J.1041.2021.00182

[58] Hu J Huang XX Wan YH Zhang SC. Research progress of mental health literacy and its influence on mental problems. Chin J Health Educ. (2022) 38:1118–22. doi: 10.16168/j.cnki.issn.1002-9982.2022.12.012

[59] Tabata-Kelly M Ruan M Dey T Sheu C Kerr E Kaafarani H . Postdischarge caregiver burden among family caregivers of older trauma patients. JAMA Surg. (2023) 158:945–52. doi: 10.1001/jamasurg.2023.2500, 37405733 PMC10323760

[60] Capobianco L Faija C Husain Z Wells A. Metacognitive beliefs and their relationship with anxiety and depression in physical illnesses: a systematic review. PLoS One. (2020) 15:e0238457. doi: 10.1371/journal.pone.0238457, 32911486 PMC7500039

[61] Fujinami R Sun V Zachariah F Uman G Grant M Ferrell B. Family caregivers’ distress levels related to quality of life, burden, and preparedness. Psychooncology. (2015) 24:54–62. doi: 10.1002/pon.3562, 24789500 PMC4216636

[62] Steele A Maruyama N Galynker I. Psychiatric symptoms in caregivers of patients with bipolar disorder: a review. J Affect Disord. (2010) 121:10–21. doi: 10.1016/j.jad.2009.04.020, 19443040

[63] Yao H Li K Li C Hu S Huang Z Chen J . Caregiving burden, depression, and anxiety in informal caregivers of people with mental illness in China: a cross-sectional survey. BMC Psychiatry. (2024) 24:824. doi: 10.1186/s12888-024-06239-4, 39563250 PMC11577895

[64] Henriksson A Carlander I Årestedt K. Factors associated with feelings of reward during ongoing family palliative caregiving. Palliat Support Care. (2015) 13:505–12. doi: 10.1017/S1478951514000145, 25994479

[65] Erkuş Ş Gümüş AB. Hope and psychological resilience in primary caregivers of patients with a chronic mental illness followed in a community mental health center. Arch Psychiatr Nurs. (2024) 50:87–93. doi: 10.1016/j.apnu.2024.03.010, 38789239

[66] Haddock G Barrowclough C Tarrier N Moring J O'Brien R Schofield N . Cognitive-behavioural therapy and motivational intervention for schizophrenia and substance misuse. 18-month outcomes of a randomised controlled trial. Br J Psychiatry. (2003) 183:418–26. doi: 10.1192/bjp.183.5.418, 14594917

[67] Kopelovich SL Stiles B Monroe-DeVita M Hardy K Hallgren K Turkington D. Psychosis REACH: effects of a brief CBT-informed training for family and caregivers of individuals with psychosis. Psychiatr Serv. (2021) 72:1254–60. doi: 10.1176/appi.ps.202000740 34015942

[68] Zhou DR Chiu YM Lo TW Lo WA Wong SS Leung CHT . An unexpected visitor and a sword play: a randomized controlled trial of collective narrative therapy groups for primary carers of people with schizophrenia. J Ment Health. (2023) 32:351–62. doi: 10.1080/09638237.2020.1793123 32667240

[69] Chien WT Ma DCF Bressington D Mou H. Family-based interventions versus standard care for people with schizophrenia. Cochrane Database Syst Rev. (2024) 10:CD013541. doi: 10.1002/14651858.CD013541.pub2, 39364773 PMC11450935

[70] Sin J Norman I. Psychoeducational interventions for family members of people with schizophrenia: a mixed-method systematic review. J Clin Psychiatry. (2013) 74:e1145–62. doi: 10.4088/JCP.12r08308 24434103

[71] Duggleby W Schroeder D Nekolaichuk C. Hope and connection: the experience of family caregivers of persons with dementia living in a long term care facility. BMC Geriatr. (2013) 13:112. doi: 10.1186/1471-2318-13-112, 24138640 PMC4015868

[72] Laranjeira CA Querido AIF Charepe ZB Dixe MDACR. Hope-based interventions in chronic disease: an integrative review in the light of Nightingale. Rev Bras Enferm. (2020) 73:e20200283. doi: 10.1590/0034-7167-2020-0283 33338171

[73] Prevo L Kremers S Jansen M. Small successes make big wins: a retrospective case study towards community engagement of low-SES families. Int J Environ Res Public Health. (2020) 17:612. doi: 10.3390/ijerph17020612, 31963678 PMC7014447

[74] Foster-Fishman PG Fitzgerald K Brandell C Nowell B Chavis D Van Egeren LA. Mobilizing residents for action: the role of small wins and strategic supports. Am J Community Psychol. (2006) 38:143–52. doi: 10.1007/s10464-006-9081-0, 17006773

[75] Chien WT Chan SW. The effectiveness of mutual support group intervention for Chinese families of people with schizophrenia: a randomised controlled trial with 24-month follow-up. Int J Nurs Stud. (2013) 50:1326–40. doi: 10.1016/j.ijnurstu.2013.01.004, 23433723

